# Fermented Food Consumption Across European Regions: Protocol for the Development and Validation of the Web-Based Fermented Foods Frequency Questionnaire (3FQ)

**DOI:** 10.2196/69212

**Published:** 2025-09-08

**Authors:** Emmanuella Magriplis, Sotiria Kotopoulou, Signe Adamberg, Kathryn Jane Burton-Pimentel, Vaida Kitryte-Syrpa, Marta Laranjo, Victoria Meslier, Theodoros Smiliotopoulos, Guy Vergères, Bojana Vidovic, Christophe Chassard, Michail Syrpas

**Affiliations:** 1 Department of Food Science and Human Nutrition Laboratory of Dietetics and Quality of Life Agricultural University of Athens Athens Greece; 2 Nutrition & Food Standards Unit Risk Assessment & Nutrition Directorate Hellenic Food Authority Athens Greece; 3 Tallinn University of Technology Tallinn Estonia; 4 Agroscope Bern Switzerland; 5 Department of Food Science and Technology Kaunas University of Technology Kaunas Lithuania; 6 Mediterranean Institute for Agriculture, Environment and Development & CHANGE-Global Change and Sustainability Institute, and Departamento de Medicina Veterinária-Escola de Ciências e Tecnologia Universidade de Évora Évora Portugal; 7 MetaGenoPolis Institut National de Recherche pour l’Agriculture, l’Alimentation et l’Environnement University Paris-Saclay Jouy-en-Josas France; 8 Faculty of Pharmacy University of Belgrade Belgrade Serbia; 9 VetAgro Sup Institut National de Recherche pour l’Agriculture, l’Alimentation et l’Environnement Université Clermont Auvergne Aurillac France

**Keywords:** fermented foods, consumption data, intake, human health, European regions

## Abstract

**Background:**

Fermented foods vary significantly by food substrate and regional consumption patterns. Although they are consumed worldwide, their intake and potential health benefits remain understudied. Europe, in particular, lacks specific consumption recommendations for most fermented foods.

**Objective:**

This project, which is under the framework of the Promoting Innovation Of Fermented Foods (PIMENTO) Cooperation in Science and Technology (COST) Action (CA20128), aims to develop a validated tool to quantitatively estimate fermented food intake across 4 European regions.

**Methods:**

The Fermented Food Frequency Questionnaire (3FQ) was designed to quantify fermented food intake in terms of frequency and quantity. Fermented foods were categorized into broad groups (eg, dairy, plant-based, meat, beverages) based on product classifications, ensuring that the foods included were genuinely fermented through ingredient analysis according to the International Scientific Association for Probiotics and Prebiotics consensus for fermented foods as a guide. For each main fermented food group, subcategories were determined after detailed discussions by a scientific expert panel that provided country-specific examples. For example, for hard cheeses, Parmigiano was chosen in the Italian version, and Graviera in the Greek version. The questionnaire was developed in English (universal version) and then translated into multiple languages using the back-translation method. Each version was pilot-tested for clarity, and data for the prospective validation were gathered. This included two key steps: (1) assessing repeatability by having participants retake the questionnaire after 6 weeks and (2) confirming accuracy by comparing 3FQ results against 24-hour dietary recalls from a subsample of participants. Statistical analyses will be used to confirm agreement between the methods. Representative sample calculations were performed for 4 groups by biological sex and age group (between 18 and 49.9 years and 50+ years). To ensure representative sample obtainment, participants aged 18+ years were recruited via the internet using multiple strategies, including social media platforms in all countries, snowball sampling, and potential supplementation with panels provided by the survey platform. Prior to all responses, participants were asked to provide informed consent and agree to data collection under ethical guidelines using a General Data Protection Regulation–compliant platform.

**Results:**

A representative sample of 1536 participants per European region was targeted, ensuring diversity in age and sex, with the goal of achieving a 60% response rate. A multilingual questionnaire was developed and pilot-tested for clarity. The upcoming steps will include final validation for accuracy and repeatability using 24-hour dietary recalls and specific statistical techniques of analysis to ensure reliability.

**Conclusions:**

The validated web-based 3FQ aims to address the current gaps in fermented food intake to help improve future research in this important area.

**International Registered Report Identifier (IRRID):**

DERR1-10.2196/69212

## Introduction

The fermentation process involves the action of microorganisms indigenously present in the food (raw) material or added as starter cultures under adequate environmental conditions [1]. In the past few years, the potential health benefits of fermented foods, microorganisms contributing to the fermentation process, and the resulting fermentation metabolites, vitamins, and bioactive compounds have raised great interest [2,3] due to their potential for improving human health [4,5] in children and adults [6]. Nevertheless, assessing the potential health benefits of fermented foods remains a challenge since over 5000 fermented foods and beverages are produced globally [7], and many remain understudied. Moreover, there are no specific recommendations for their consumption in Europe [8] since specific consumption data are limited and addressed mainly in overall dietary assessments.

It is important to consider the variety of substrates, products, and microorganisms involved to understand the diversity of fermented foods and beverages [9]. For instance, although the same substrate, milk, can be the basis for most of the available fermented dairy products (eg, yogurt, kefir, and cheese) [10], the final products differ considerably in terms of nutritional composition and bioactive compounds. Additionally, the fermentation process may involve the action of one or more microorganisms that are either indigenously present in the raw material, added as starter cultures, or introduced via back-slopping and grown under appropriate environmental conditions that may or may not be alive at the time of consumption [1,11]. Moreover, the microorganisms used for fermentation are associated with different metabolic pathways and reactions involved in the process, leading to various physicochemical transformations in the final products. For example, different groups of microbes may produce different end products (metabolites), including organic acids (eg, lactic, acetic acid) through various metabolic pathways under different conditions [12]. Additionally, while some microorganisms are known for their capacity to synthesize specific vitamins (folate, riboflavin, and cobalamin) from various precursors in plant and dairy foods [13], others do not influence or even consume vitamins [14]. Furthermore, various fermented foods have been reported to contain a series of bioactive peptides derived through the action of proteases synthesized by fermenting microorganisms [8]. Consequently, it is not surprising that fermentation from a nutritional perspective is also associated with the reduction of antinutritional factors and an improvement in the overall nutritional value and digestibility of fermented foods [15].

Despite their potential health benefits, fermented foods are generally not recommended as a category in dietary guidelines; in fact, there are no specific recommendations for their consumption in Europe [8,13]. A challenging factor is the fact that the type of fermented food consumed varies by region and country [8]. Differentiation by main food substrate is necessary to understand and assess the potential health benefits of fermented foods and acquire ample information to make specific recommendations. As aforementioned, these food substrates may vary significantly regarding nutritional composition and products formed during fermentation; therefore, population-based consumption data are necessary to understand their role in human health.

To date, there remain limited quantification methods specifically developed for assessing fermented food intake in populations. A recent study identified and estimated fermented foods consumed in Japan using dietary recalls by season and found 1396 unique fermented foods consumed [16]. In the Dutch observational cohort (NQplus) that used a food frequency questionnaire (FFQ), an estimated 17% of foods consumed were fermented, and another 13% were included in composite dishes [17]. The validation results showed that FFQs could effectively estimate foods and beverages that are regularly consumed, such as coffee, bread, and cheese, since the mean difference between recalls and the FFQ was small. However, this was not the case for fermented foods consumed more sporadically [17]. Both studies support the hypothesis of the variety of fermented foods available, regardless of the food substrate, and highlight the importance of developing a tool to assess the intake of these food substrates and their main byproducts. This represents a crucial first step toward understanding their effects on human health.

In this context, another recent study evaluated food intake biomarkers for various fermented foods to discriminate the dietary patterns of fermented foods [18]. Although this is of great value, many biomarkers could be affected by human metabolic rates (concentration biomarkers) and therefore do not accurately reflect absolute food intake. Therefore, estimating frequency and quantifying fermented food intake using validated dietary assessment methods remains necessary [19], along with developing tools that minimize their potential limitations [20].

One of the main limitations is accurate quantification, which can be addressed using validated color food atlas pictures, where each icon refers to a specific portion size (by weight), allowing the individual to select their usual portion from a series of pictures rather than relying on subjective quantity estimates [15]. This process [21], combined with using food models or selected house measures when no valid food picture is available, has been found to increase intake accuracy [22]. Nevertheless, because fermented foods represent a specific type of food intake, a tool that explicitly evaluates their consumption—providing specific examples by substrate and potential subcategories—can help overcome the limited information reported for these foods. Assessing intake in a representative population may help validate types of biomarkers for further research on fermented foods and health.

The primary aim of this questionnaire is to collect and analyze data on fermented food consumption, using predefined food groups and their substrates, in the 4 main European regions, as defined by EuroVoc [23]. The project is carried out as part of the Promoting Innovation of Fermented Foods (PIMENTO) Cooperation in Science and Technology (COST) Action (CA20128) [24], with the aim of creating a universally validated tool for recording the intake of fermented foods among European consumers.

## Methods

### Ethical Considerations

This research protocol was approved by the Ethics Committee of the Agricultural University of Athens (27/05.05.2023). The study process abides by the ethical principles for research involving humans, as reported by the Declaration of Helsinki [25].

### Identification and Classification of Fermented Foods

Identifying and classifying fermented foods can be challenging because there are multiple definitions and perceptions of what constitutes a fermented food. To address this, in 2019, an expert panel by the International Scientific Association for Probiotics and Prebiotics defined fermented foods as “foods made through desired microbial growth and enzymatic conversions of foods” [23]. This definition encompasses a wide range of products that may or may not contain live microorganisms and is the basis of the definition used for this questionnaire.

Considering the wide variety of fermented foods available in the marketplace and consumed by different European populations, fermented foods were first stratified in the Fermented Food Frequency Questionnaire (3FQ) into broad food groups: plant-based products, dairy products, legumes, meat and/or fish, vegetables, cereal products, chocolate, nonalcoholic beverages, vinegar, coffee, tea, chocolate beverages, beer and/or cider, wine, and spirits. These food groups were defined a priori, based on the classification of product categories used for mapping fermented foods within the activities of Working Group 2 from the PIMENTO CA20128.

Fermented foods within each food group were then aggregated into subgroups. A series of exclusion criteria was also applied to ensure that the studied foods were indeed fermented. Foods that were traditionally fermented but are no longer fermented because of contemporary food processing (such as pickled vegetables) or foods that were partially fermented (such as green or black teas that are typically oxidized rather than post fermented) were included or eliminated based on the ingredient lists of commonly consumed grocery store products. With the exception of chocolate and some plant-based alternatives, partially fermented foods (ie, salad dressings) and composite dishes (ie, chocolate-based confectionery) were not considered. For example, sauerkraut is made from cabbage, water, and salt and its sour taste and preservation are achieved through lactic acid fermentation by live bacteria, which aligns with this study's definition of a fermented food, whereas pickled gherkins made from cucumbers, water, vinegar, salt, and sugar get their sourness and acidity directly by the added vinegar, not by microbial activity. Therefore, this product was excluded as it is pickled but not fermented.

### Study Sample

The study aimed to include a representative sample for each of the 4 European regions. A predefined sampling frame was formulated, and the World Health Organization STEPwise procedure was followed. The minimum sample size required for each European region was calculated to achieve 80% study power at a 95% confidence level. A response rate of 60%, as reported by many epidemiological studies, was accounted for, along with a potential 10% attrition rate. The goal was to achieve a representative sample for 4 groups: males and females, young adults (between 18 and 50 years), and older adults (≥50 years of age). Based on a 5% margin of error and a conservative approach of 50% for the indicators to be assessed, a total sample of 1536 from each European region was required (for 4 groups). This translated to a target of 2560 contacts, based on the 60% anticipated response rate. The representativeness by group was monitored (quota monitoring) throughout the study process based on European Region population distribution response rates. In situations where representativity for certain quotas (region, age group, sex) was not achieved, the web-based platform selected provided the option to use predefined panels from specified areas and required characteristics. Specifically, the platform could be used to target sampling from predefined panels in underrepresented areas and specific age groups. The predefined panels could also be used to rectify imbalances in the sex distribution, as evaluated during the development of the sampling frame and assessment of representatives for each European region. Responses obtained through this method were integrated with the originally collected data, ensuring a cohesive and representative final data set that aligns with our study aims. However, sensitivity analyses will be conducted (with and without the extra data from the panels) to evaluate potential response differences that can also impact the results. Initially, a comprehensive comparison of the essential demographic, socioeconomic, and lifestyle attributes of participants recruited from the internet versus those from predetermined panels will be performed to assess whether there are substantial variations in parameters that could affect the primary outcomes—documented consumption patterns of fermented foods—between the 2 groups. A primary analysis using the complete, integrated data set will be performed, followed by a secondary analysis using exclusively the nonpanel sample. The 2 results will be compared to ascertain whether the incorporation of panel data significantly modifies the study's conclusions. The outcomes of these sensitivity studies will be fully disclosed in the publication.

### Inclusion/Exclusion Criteria

Individuals were considered eligible for participation in the study when they were above 18 years of age at the time of recruitment and agreed to sign the informed consent form. The survey was conducted over the internet, and the questionnaire was hosted on the Conjointly platform. Participants were recruited through the use of many channels: (1) the PIMENTO website [26]; (2) social media (ie, LinkedIn, Facebook, X, Instagram); (3) emailing past survey participants for whom we have contact information; (4) through information from notified sites and nonprofit organizations; and (5) through snowball sampling where respondents were encouraged to forward the invitation to any interested parties.

Conjointly is a web-based research platform used in this study to recruit participants and administer the 3FQ. It is General Data Protection Regulation (GDPR) accredited and offers an option not to maintain IP addresses, thereby increasing the study’s confidentiality. It was chosen since it provides researchers with integrated tools to build and customize surveys using a wide range of question formats, from simple scales to complex experimental designs. It also generates QR codes automatically and is accessible through smartphones (iOS and Android). The platform codes responses automatically as uploaded, and it provides the total database coded and with summary statistics for rapid data evaluation during the recruitment phase and sample data collection, reducing selection bias. It also supports advanced analytical methodologies, which are used to statistically measure consumer preferences.

Each participant was asked to provide informed consent before proceeding with the survey. The informed consent included a detailed explanation of the study's main aim, including how data would be collected, stored, and used, and the potential risks associated with web-based questionnaires. It also underlined that the participants are not obliged to respond to all questions and that they can leave the study with no consequences at any time. For the validation process (second part of the study), which asked for personally identifiable information, participants were required to provide consent again in order to be contacted. Additionally, participants selected from predefined panels participated in the primary survey only and were ineligible for the validation phase of the 3FQ, thus segregating the validation outcomes from this possible source of bias. The study protocol has been preregistered in the Open Science Framework, a registry that accepts observational studies as recommended to help decrease publication bias [27].

### Study Questionnaire

Respondents were initially asked to answer general questions, including self-reported anthropometric data. They also answered basic questions regarding their health state and any allergies, as these parameters may affect knowledge and behavior related to fermented food consumption. Participants were then presented with the 12 fermented food categories and asked to choose whether they consume each of these food categories and, through visualizations, report their usual consumption portion. The included fermented food categories were plant-based meat alternatives, dairy products, legumes, meat, fish, vegetables, cereal products, chocolate (bars), nonalcoholic and alcoholic beverages (beer and/or cider, wine, and spirits), vinegar, and beverages (coffee, tea, and chocolate beverages). Although this classification is rather extensive, as discussed earlier, some food groups included in the 3FQ structure may not be widespread or frequently consumed in all countries, which is the main challenge as well as the necessity of this multi-European region study to identify differences in the amount and type of fermented foods consumed in each region.

### Questionnaire Development and Validation

The questionnaire was first developed in English by experts in food science and technology, nutrition, nutritional epidemiology, and consumer science. Up to 2 National Contact Points (NCPs) from each country were responsible for translating the questionnaire into their respective languages, ensuring it matched the originally derived international tool (English version). However, each country used nationally available food examples per main food group question to personalize the questionnaire (eg, in the cheese group, Greece used feta and Italy used mozzarella as soft/semisoft cheese examples). Countries with more than 1 national language translated the questionnaire in all (eg, Switzerland translated it into French, Italian, and German, along with other national translations from the respective countries). A standardized translation methodology was followed, which included two steps: (1) translation to the national language by an individual fluent in both the national and English languages and (2) back-translation from the national language to English, with the individual responsible for the second step being blinded to the primary international questionnaire. The back-translated questionnaire was compared to the international questionnaire, and necessary corrections were made.

The clarity of the questionnaire was assessed through pilot testing. This was performed through the Conjointly platform, using a sample of 50 individuals from various age groups and educational backgrounds. The participants were asked to note any difficulties they faced when accessing or responding to the questions and/or potential clarifications required. These were reviewed by the NCP members, and corrections were included in the final version.

The 3FQ will also undergo validation, a process that includes 2 parts. The first part aims to ensure the repeatability of the 3FQ across all European regions, and the second part assesses overall accuracy. For repeatability, participants were asked if they would be willing to repeat the same questionnaire approximately 6 weeks after their first response. Those who agreed were asked to provide consent and provide an email address and or phone number for further contact at a later stage. A minimum of 200 responders from each European region was calculated as necessary to meet the commonly used rule of thumb of 5 respondents per question.

The second part of the validation will assess the accuracy of the 3FQ using a harmonized methodology. This process will involve participants from the total sample across the participating countries. To ensure a harmonized process, NCPs who agreed to act as interviewers were trained by a dietitian specializing in nutritional research methods through two 2-hour web-based sessions and 1 on-site workshop. A specific Excel (Microsoft Corp) file using drop-down menus was developed and shared with all interviewers to be used during the recall process for each individual.

For the actual procedure, participants were asked if they agreed to share their communication details (as previously described) and to be contacted for a web-based interview about their usual dietary habits. Individuals who shared either a phone number or email address were contacted no later than 2 weeks after completing the web-based 3FQ to undergo a 24-hour phone-based recall. The validation process included one or two 24-hour phone-based recalls, using the Automated Multipass Method, from a representative sample of the study’s target population [28]. A total of 265 to 371 individuals were required for this process, as per the rule of thumb (between 5 and 7 participants per survey question).

The study’s power calculation was performed for the Spearman correlation analysis using G*Power software (Heinrich Heine University Düsseldorf). Specifically, the sample size needed to achieve 90% power, assuming a low correlation of ρ_ο_=0.4 (the null hypothesis H_o_) with the potential for no correlation (the alternative hypothesis H_1_=0.2), at α =5% (2-tailed exact test), was calculated to be 218 participants. Since only 1 recall was expected from most participants, and because Bland-Altman analysis requires more data, a larger sample based on the 7 participants per question rule was targeted to reduce random error. The higher number (371) was chosen as the optimal target, as it ensures adequate power to assess the accuracy of the subproducts of each food group.

Participants who did not provide dietary assessment data or complete at least 1 recall were excluded from the validation data set. At least 1 recall was required (and up to 2 were performed) for the validation of the 3FQ’s accuracy. This criterion was chosen since the aim was not to assess overall usual intake but to validate the 3FQ through a meaningful measure of fermented food intake. Permitting the collection of recalls on 1 or 2 days can help reduce participant burden, thereby decreasing selection bias among working individuals. Additionally, it was clearly communicated that the recall would be interview-based to help reassure and decrease selection bias among older participants, who are generally more hesitant with web-based processes.

Moreover, measures were taken to ensure a representative sample for the 3FQ responses, specifically including an older age group that was 50+ years, for representativeness of the 3FQ responses. For the questionnaire’s validation, demographics will be disclosed to depict the population to which the results may be justifiably generalized. After merging the 3FQ and 24-hour recall data, the population sample will represent the validation subsample for further analyses.

Data on fermented foods from the 3FQ and recalls will be identified and aggregated into conventional food groups. Percent differences in mean intakes, quintile cross-classification, Spearman correlations, and Bland-Altman analyses will be used to evaluate the agreement between the 2 dietary assessment methods.

## Results

This project is part of the PIMENTO COST Action (CA20128) but did not receive specific funding, other than member travel and accommodation costs during on-site working groups. The questionnaire has been developed, translated, and pilot-tested, with the main data collection phase beginning in October 2023 and concluding in May 2024. The validation phase, which will include repeatability testing and 24-hour dietary recalls, was completed in December 2024. Data analysis was set to start in January 2025, with the results anticipated for publication 4 months after the analysis commences, estimated in September 2025.

## Discussion

### Overview and Important Considerations

The study project handles a variety of operational, methodological, and ethical concerns, particularly in relation to the use of web-based surveys. A significant limitation is the exclusion of susceptible demographics like children, adolescents, and individuals without legal capacity, reducing the generalizability of the results. Furthermore, there are advantages and disadvantages to performing web-based surveys. An important advantage that is an asset pertaining to the study's aim is the ability to reach a sizable, geographically diversified sample at a reasonable cost. Web-based surveys also offer flexibility, allowing respondents to finish the survey at their convenience. The GDPR-certified platform enables automated data collecting and processing, helping minimize human error and misclassification.

However, internet surveys have certain drawbacks that must be considered, such as selection bias. For example, people without internet access or low technological proficiency may not be included, resulting in the underrepresentation of certain demographic groups. Furthermore, response rates might be lower than in-person techniques due to possible difficulties in understanding the question.

To address these challenges, different forms of pilot testing were conducted before the study launch. First, the survey was shared with a panel of food science researchers and nutritionists, who completed it and provided feedback on areas requiring correction and/or clarification. The feedback received was reviewed by the study's coprincipal investigators, discussed with the NCPs, and used to make improvements/adjustments. Next, each NCP also conducted a pilot phase using a convenience sample from the general population in their country. Issues encountered during this phase were discussed, and further adjustments were made to improve clarity.

Privacy issues pertaining to the management of anthropometric, health, and personal data are also of great importance, especially when creating profiles. Data will be anonymized and securely stored, with restricted access to authorized personnel. Only the absolutely necessary anonymized personal data will be stored for a maximum of 4 years at the Agricultural University of Athens. After this period, the principal investigators will carefully dispose of the data.

In cases where predefined panels were used because representativity and certain quotas were not met, sensitivity analyses will be conducted. Any significant differences between the 2 groups (based on the collection method) found from comparisons and tests performed will be highlighted. If the results are consistent across groups, this will strengthen the study’s validity. If the results differ, the data will not be combined; instead, the results will be separately presented, and a detailed discussion on the implications will be derived as necessary. A weighted approach to balance the influence of each data source may be used if differences are minimized. The results will be ethically handled and disseminated to safeguard participant confidentiality while effectively informing interested parties, including the public, academics, and policy makers. Finally, despite the challenges recognized, careful planning and ethical supervision are in place to ensure that the study significantly contributes to the field of nutrition and food science, paving the way for necessary future actions.

### Limitations

While we cannot fully eliminate the potential for social desirability bias, especially for foods perceived as “healthy,” the methodology used aimed to mitigate reporting errors. Specifically, the Automated Multipass Method for the 24-hour recalls was used to minimize misreporting and improve recall accuracy through systematic probing, and the 3FQ was designed with a hierarchical structure that included broad food groups followed by specific fermented subgroups. This was used as a “filter” to systematically guide participants' responses and help reduce perception errors.

Furthermore, fermented foods like kefir, kombucha, and kimchi are distinctive and often consumed intentionally or due to cultural reasons. This may lead to more accurate reporting compared to more commonly consumed items.

The potential underrepresentation of individuals with lower literacy levels or those without computer or internet access should also be considered. The platform selected was accessible through a smartphone—either Android or iOS—and easily accessible through a QR code, potentially mitigating the selection bias of individuals with no computer access.

Regarding the effect of literacy levels, it is plausible that the subject matter itself—fermented foods consumption—may have an inherent selection effect towards a more literate population, hence potentially reducing the bias introduced by a web-based survey tool compared to a survey on a more general topic. Future analyses could address this by statistically weighing the data against national census statistics using demographics on literacy levels. This information has been included in the Methods section (where the Conjointly platform is described) and the limitations section (for the literacy level and prospective possibilities).

### Conclusion

The web-based 3FQ is designed to address existing research gaps in assessing the frequency and quantity of fermented food consumption across all food groups. This will enable researchers to gain deeper insights into dietary patterns involving fermented foods and support more robust analyses of their potential associations with health outcomes, while also enhancing the accuracy and comparability of data in future studies. This tool is also capable of addressing consumption data of less frequently consumed fermented foods that are regularly missed in usual food frequency questionnaires, thereby minimizing systematic consumption errors.

## Abbreviations

3FQ: Fermented Foods Frequency Questionnaire
COST: Cooperation in Science and Technology
FFQ: food frequency questionnaire
GDPR: General Data Protection Regulation
NCP: National Contact Point
PIMENTO: Promoting Innovation of Fermented Foods

## Data Availability

The study protocol has been uploaded to the Open Science Framework [29]. Data collected will also be included and open after an embargo period of 3 years. Any database used for publication will be made available, without details on individual patient data, after manuscript acceptance.
