# Correction: Fermented Food Consumption Across European Regions: Protocol for the Development and Validation of the Web-Based Fermented Foods Frequency Questionnaire (3FQ)

**DOI:** 10.2196/86393

**Published:** 2025-12-29

**Authors:** Emmanuella Magriplis, Sotiria Kotopoulou, Signe Adamberg, Kathryn Jane Burton-Pimentel, Vaida Kitryte-Syrpa, Marta Laranjo, Victoria Meslier, Theodoros Smiliotopoulos, Guy Vergères, Bojana Vidovic, Christophe Chassard, Michail Syrpas

**Affiliations:** 1 Department of Food Science and Human Nutrition Laboratory of Dietetics and Quality of Life Agricultural University of Athens Athens, Attica Greece; 2 Nutrition & Food Standards Unit Risk Assessment & Nutrition Directorate Hellenic Food Authority Athens Greece; 3 Tallinn University of Technology Tallinn Estonia; 4 Agroscope Bern Switzerland; 5 Department of Food Science and Technology Kaunas University of Technology Kaunas Lithuania; 6 Mediterranean Institute for Agriculture, Environment and Development & CHANGE-Global Change and Sustainability Institute, and Departamento de Medicina Veterinária-Escola de Ciências e Tecnologia Universidade de Évora Évora Portugal; 7 MetaGenoPolis Institut National de Recherche pour l’Agriculture, l’Alimentation et l’Environnement University Paris-Saclay Jouy-en-Josas France; 8 Faculty of Pharmacy University of Belgrade Belgrade Serbia; 9 VetAgro Sup Institut National de Recherche pour l’Agriculture, l’Alimentation et l’Environnement Université Clermont Auvergne Aurillac France

In Fermented Food Consumption Across European Regions: Protocol for the Development and Validation of an Web-Based Fermented Foods Frequency Questionnaire (3FQ)” [1] the authors noted one error which was an omission to acknowledge the work from COST Action.

A correction of the Acknowledgements section adding the following text:

This article/publication is based upon work from COST Action PIMENTO CA20128, supported by COST (European Cooperation in Science and Technology).”

The Acknowledgments section now reads:

This article/publication is based upon work from COST Action PIMENTO CA20128, supported by COST (European Cooperation in Science and Technology).

The authors sincerely thank all National Contact Points (NCPs) who helped disseminate the questionnaire (in alphabetical order): K Adamberg, D Ağagündüz, M Beglaryan, D Borch Ibsen, E Brouwer Brolsma, J Burtscher, M Cerjak, K Chandolias, Z Ciesarová, I Ciproviča, De Filippis, E Delia, M Deschasaux, A Gamero, M Gandia, A Giertlova, MC Grech Perry, L Hoxha, D Jaros, H Jenssen, P Jones, A Kalea, B Kalyoncu, J Karl, L Kennes, G Kostov, A Kusar, M Laranjo, E Mantzari, N Mikulec, E Mudura, NE Nagybakay, L Ove Dragsted, L Ozola, NA Pancevska, P Papademas, D Pipoyan, I Pravst, H Rohm, P Russo, T Sar, M Starowicz, I Talkic, B Trajkovska, A Vukojevic, M Wronkowska, G Nakov, T Pohjanheimo. The authors received financial reimbursement from the Promoting Innovation of Fermented Foods (PIMENTO) Cooperation in Science and Technology (COST) Action (CA20128) for the meetings and workshops conducted during tool preparation. Author MS received a grant to launch the questionnaire to specific populations. Author SK is alone responsible for the content and views expressed in this study, and they do not represent the decisions, policy, or views of the Hellenic Food Authority.

The correction will appear in the online version of the paper on the JMIR Publications website, together with the publication of this correction notice. Because this was made after submission to PubMed, PubMed Central, and other full-text repositories, the corrected article has also been resubmitted to those repositories.

## References

[1] Magriplis  E, Kotopoulou S, Adamberg S, Burton-Pimentel K J, Kitryte-Syrpa V, Laranjo M, Meslier V, Smiliotopoulos T, Vergères G, Vidovic B, Chassard C, Syrpas M (2025). Fermented food consumption across European regions: protocol for the development and validation of the web-based Fermented Foods Frequency Questionnaire (3FQ). JMIR Res Protoc.

